# Supplementary material for: Temporal regularities shape perceptual decisions and striatal dopamine signals
Source: Nat Commun. 2024 Aug 17;15:7093. doi: 10.1038/s41467-024-51393-8 (PMC11330509; doi:10.1038/s41467-024-51393-8)
Supplement: Supplementary file 1 — Supplementary Information [file 41467_2024_51393_MOESM1_ESM.pdf]

Supplementary Figures

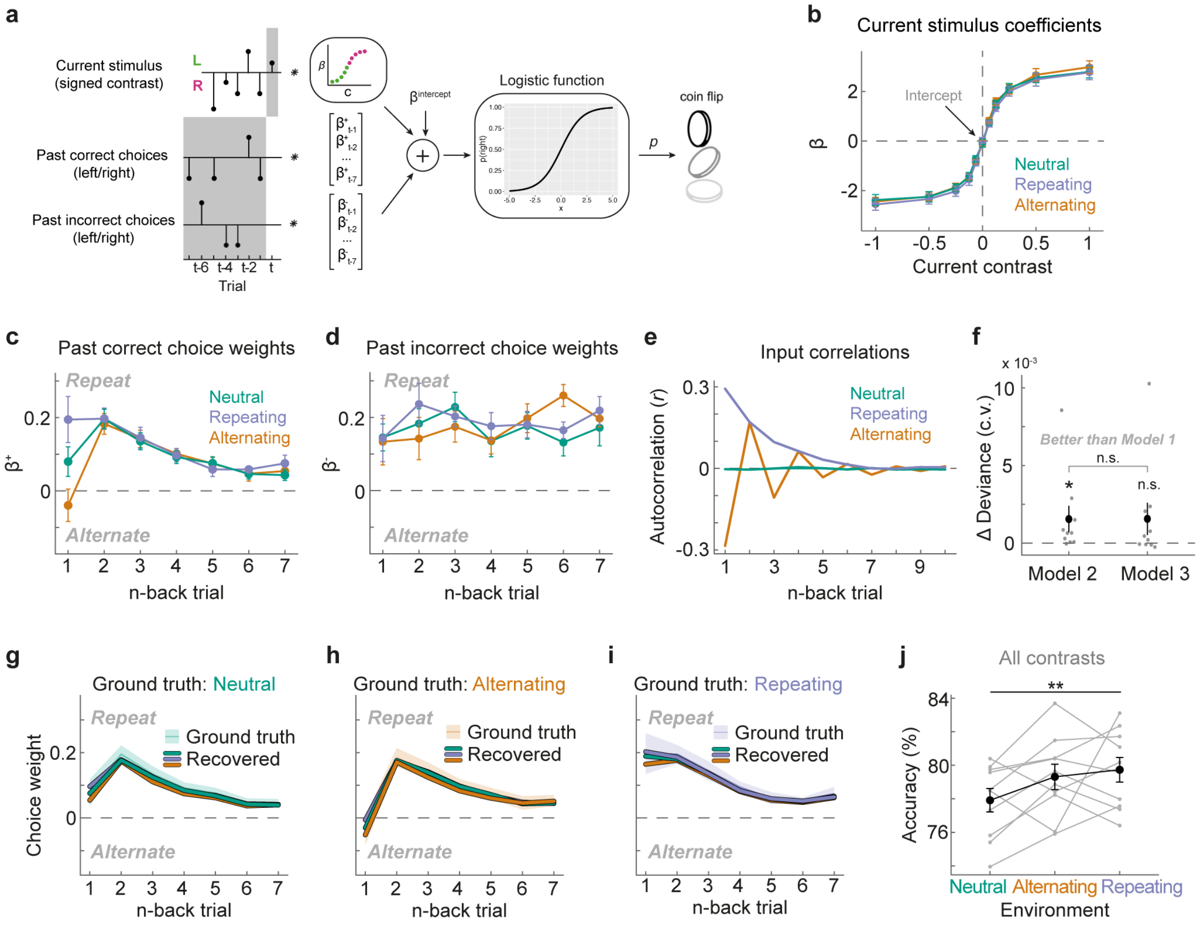

Supplementary Fig. 1 | Weights of the probabilistic choice model for different temporal regularities, parameter recovery analysis of choice history kernels, and behavioral accuracy across environments.

a, Illustration of the probabilistic choice model. We modeled the probability of the mouse making a rightward choice as a weighted sum of the current trial's sensory evidence, the successful and unsuccessful response directions of the past seven trials and a general bias term, passed through a logistic link function. Adapted from Busse, L. et al. The Detection of Visual Contrast in the Behaving Mouse. J. Neurosci. 31, 11351–11361 917 (2011). 10.1523/JNEUROSCI.6689-10.2011. https://creativecommons.org/licenses/by/4.0/ **b,** Weights associated with the current stimulus. Mice (*n* = 10) were tuned to the current stimulus, assigning negative and positive weights to left and right stimuli, respectively. The weighing of the current stimulus was similar across environments. **c,** Weights of successful choices of the past seven trials. Same as **Fig. 2a**. **d,** Weights of unsuccessful choices of the past seven trials. **e,** Autocorrelation of the empirical stimulus sequences in the three different environments. The autocorrelation approaches zero for lags larger than seven trials back. Therefore, we limited the probabilistic choice model to the past seven trials. **f,** Comparison of cross-validated binomial deviance of a probabilistic choice model with flexible 1-back weight (Model 2) and a model with flexible 1- and 2-back weights (Model 3) versus a model with fixed 1-back weight across environments (Model 1). Model 2 provides a significantly better fit compared to Model 1 (one-sided paired t-test, $t(9) = 1.88$, $p = 0.045$). **g,** The postulated ground truth choice history kernel of the neutral environment (green shaded area) was successfully recovered from simulated responses to neutral, repeating and alternating stimulus sequences (green, blue and orange lines; see Methods). **h** and **i**, same as in panel a but for postulated alternating and repeating ground truth history kernels. For each ground truth parameter set, we simulated 100 synthetic response sequences per mouse. The recovered history kernels show the average across all 100 simulations and mice. **j,** Behavioral choice accuracy across all contrasts in the three different environments. Mice exhibited a gain in performance in the

repeating and alternating over the neutral environment (two-sided paired t-test, t(9) = 4.00, p = 0.003). Gray and black lines depict individual mice and the group average. Error bars in all panels depict SEMs.

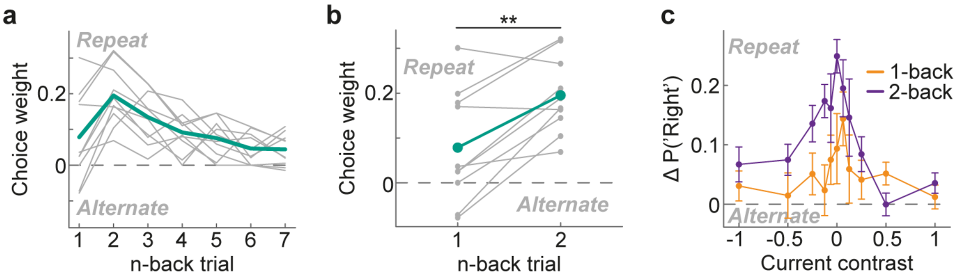

Supplementary Fig. 2 | Individual choice history kernels in the neutral environment and a model-free comparison of 1- vs- 2-back choice repetition biases.
a, History kernels comprising the past seven successful choice weights of the probabilistic choice model in the neutral environment (n = 10). **b,** Same as in panel a, but focused on individual 1- and 2-back correct choice weights. Mice were more likely to repeat their 2-back choice compared to the more recent 1-back choice. **c,** A model-free comparison between 1- and 2-back choice repetition biases. From the raw response data, we computed the difference between choice probabilities conditioned on the 1- and 2-back successful choice (right minus left), yielding ΔP('Right'). Positive values of ΔP('Right') indicate a tendency to repeat the n-back choice. The average value of ΔP('Right') across all contrast levels was significantly higher when conditioning on the 2-back versus the 1-back trial (t(9) = -3.49, p = 0.007, two-sided paired t-test). This indicates that mice were more likely to repeat their 2-back choice compared to the more recent 1-back choice when acting on random stimulus sequences, congruent with the logistic regression analysis.

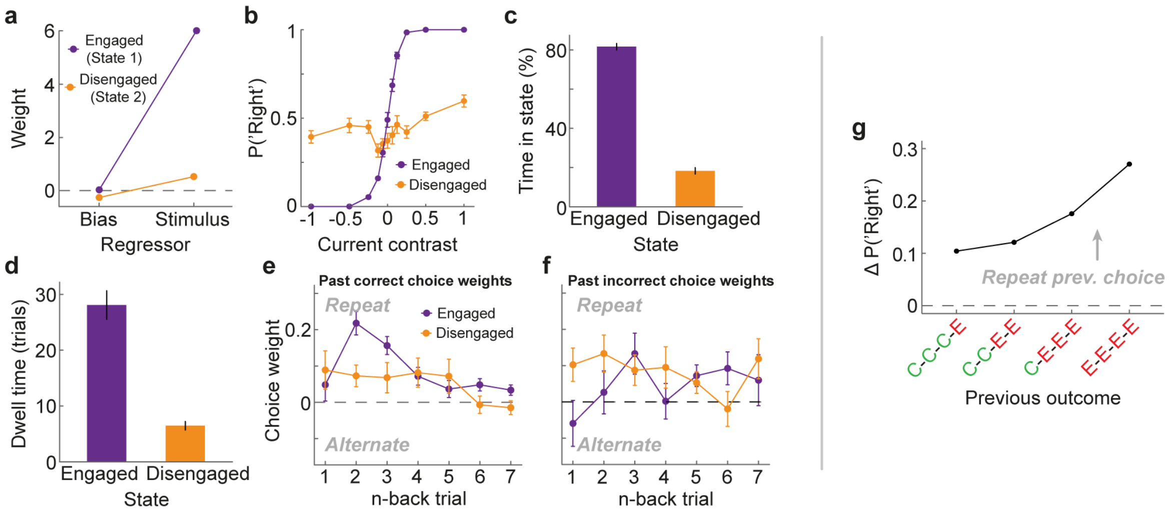

Supplementary Fig. 3 | Hidden Markov Model reveals distinct choice history kernels in engaged versus disengaged states.
a, A two-state GLM-HMM reveals states with high stimulus weight (purple, state 1, engaged) and low stimulus weight (yellow, state 2, disengaged). **b,** Psychometric curves of trials in engaged and disengaged states. Error bars in all panels depict SEMs. **c,** Mice spent considerably more time (proportion of trials) in the engaged than disengaged state (81.7 vs. 18.3%), but the proportion of disengaged trials was non-negligible. **d,** Dwell time in each state, defined as the average number of consecutive trials spent in the respective state (engaged: 28 trials; disengaged: 6.5 trials). **e,** Choice history kernel for past correct trials, separately for engaged (purple) and disengaged trials (yellow). The 1- to 2-back increase in choice weights is only present in engaged trials, while mice (n = 10) show a constant repetition bias in disengaged trials. **f,** Choice history kernel for past incorrect trials. Mice tend to alternate the 1-back incorrect choice in engaged trials, but show a constant repetition bias for disengaged trials. **g,** The choice repetition bias after an incorrect trial increased when the 1-back error

was preceded by multiple previous error trials, indicating periods of task disengagement. This further corroborates the hypothesis that the repetition of previous unsuccessful choices is, at least in part, driven by periods of task disengagement in which mice entirely ignore visual stimuli and repeat the same choice for a number of trials. The analysis in panel g is based on data from 99 mice[20].

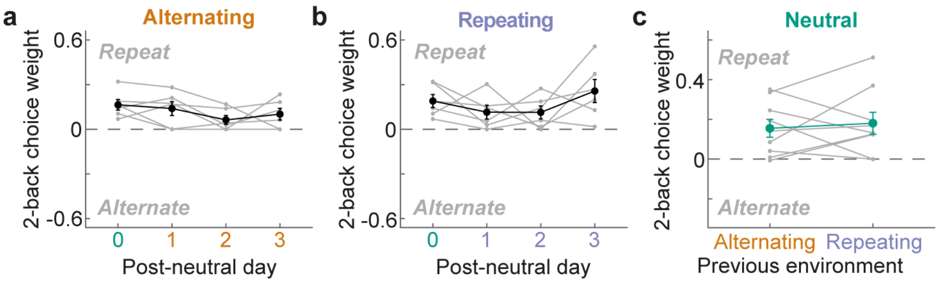

Supplementary Fig. 4 | Evolution of 2-back choice weights across days.
a, 2-back successful choice weights estimated on the first, second and third day of alternating sessions following a neutral session (*n* = 10 mice). The 2-back weight did not consistently change across post-neutral sessions (no main effect of post-neutral day in repeated-measures ANOVA, $F(2,10) = 1.22$, $p = 0.336$) **b,** Same as in panel a, but for repeating sessions ($F(2,10) = 2.028$, $p = 0.182$). **c,** 2-back successful choice weight of neutral sessions preceded by repeating or alternating sessions in each mouse (gray lines) and across the population (green line). The 2-back choice weight did not depend on the regularity of the previous session ($t(8) = 0.47$, $p = 0.65$, two-sided paired t-test).

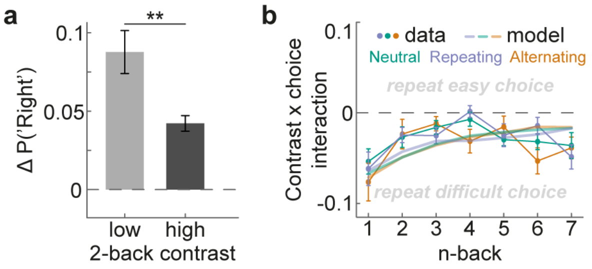

Supplementary Fig. 5 | Modulation of choice repetition bias by 2-back and n-back sensory uncertainty.
a, Difference in choice probabilities conditioned on the 2-back trial's successful choice, split according to whether the 2-back trial's stimulus contrast was high (black) or low (gray), similar to **Figure 2h** (1-back contrast). Mice (*n* = 10) are more likely to repeat the 2-back choice when it was based on a low rather than high contrast stimulus. $^{**}p<0.01$, two-sided paired t-test, $t(9) = -3.67$, $p = 0.005$. **b,** Coefficients of interaction between n-back successful choice and n-back contrast in the probabilistic choice model (colored points). Negative coefficients indicate a tendency to repeat a choice based on low rather than high stimulus contrast. The modulation by the modulation by contrast is similar across environments and decays gradually over n-back trials, which is recapitulated by the multi-trial POMDP RL model (thick colored lines).

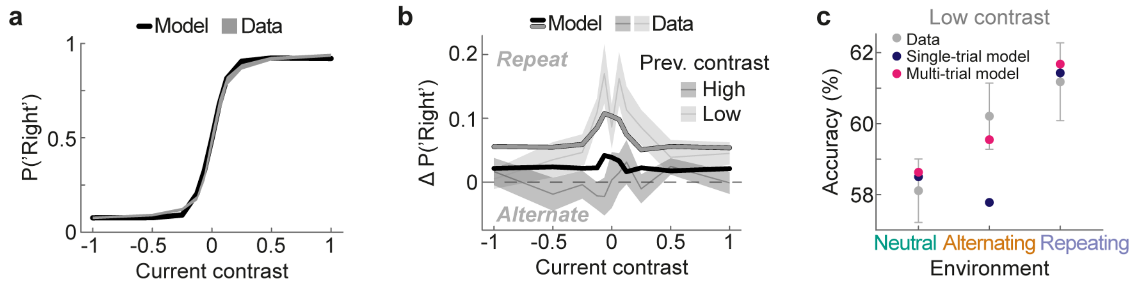

Supplementary Fig. 6 | Predictions of the single-trial belief state model.
a, Psychometric curves of the mice (shaded region, mean ± SEM, *n* = 10), and the multi-trial model (line). The model accurately captures the mice's dependence of choice (*y* axis) on current contrast (*x* axis). **b,** Difference in choice probabilities conditioned on the previous trial's successful choice split according to whether the previous trial's stimulus contrast was high (black) or low (gray). The model (lines) captures the mice's higher tendency to repeat the previous choice when it was based on a low rather than high contrast stimulus (black and gray shaded regions). **c,** Choice accuracy of the single-trial (blue) and multi-trial belief state models (pink), and the mice's empirical choice accuracy (gray). The single-trial model fails to capture the empirical increase in choice accuracy from the alternating compared to neutral environment.

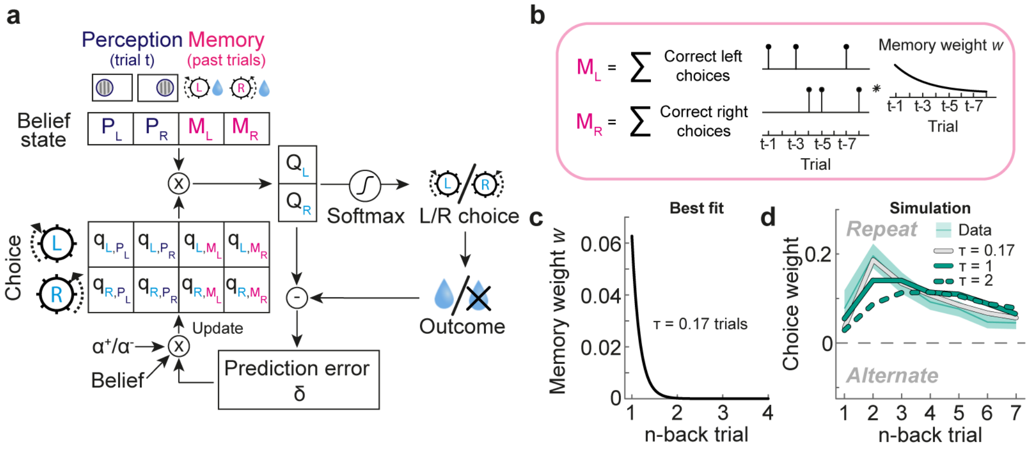

Supplementary Fig. 7 | Multi-trial reinforcement learning model with extended memory for past choices.
a, The multi-trial belief state model considers its belief about the current visual stimulus (top; dark blue), and a memory of rewarded choices made in the recent past (top; pink). **b,** Memory of recent rewarded choices is computed as an exponentially weighted sum of past left and right rewarded choices, with the most recent choice exerting the strongest influence. **c,** The memory decay of the best fitting model was quick, such that memory was limited to the 1-back trial. **d,** Simulated models with slower memory decay (τ = 1 and 2) exhibited reduced 2-back choice repetition biases, in contrast to the empirical data and best fitting model (τ = 0.17).

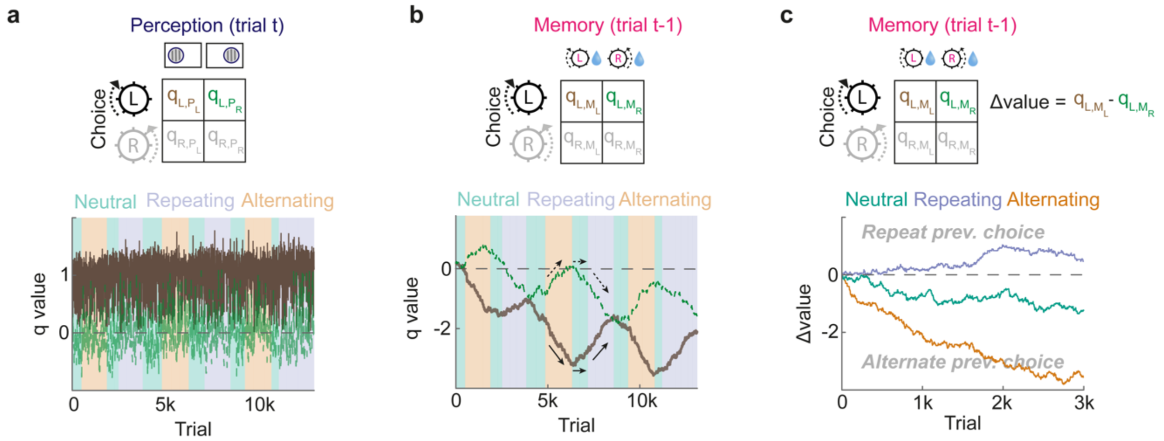

Supplementary Fig. 8 | Trial-by-trial dynamics of perception-choice and memory-choice values in the multi-trial belief state model.

a, Trial-by-trial value estimates of the multi-trial model for performing a left choice when the current stimulus is presented on the left (brown) or right side (green), across sessions with different temporal regularities (background color). The estimated value of performing the correct choice (i.e., left choice for left stimulus) approaches the true reward value (here 1), while the value estimate for the incorrect choice approaches zero. Nevertheless, value estimates rapidly fluctuate from trial to trial, due to uncertainty-weighted updating. **b,** Trial-by-trial memory-choice values in the same trials as in panel a. The value of performing a left choice after a previous successful left choice (i.e., repeating successive choices; brown) decreases in the alternating environment and increases in the repeating environment (small arrows). The value of performing a right choice after a successful left choice (i.e., alternating successive choices; green) develops oppositely. The diverging memory-choice values in the alternating environment promote an alternation bias away from the previous successful choice, which is slowly reverted in the repeating environment via converging memory-choice values. **c,** Difference in memory-choice values (Δvalue; prev. successful choice left minus right) for a multi-trial model operating on a neutral (green), repeating (blue) or alternating (orange) stimulus sequence. The model develops negative Δvalue estimates in the neutral and alternating environments, promoting alternations from previous successful choices, thereby counteracting maladaptive choice repetition biases driven by perception-choice learning. In the repeating environment the model develops slightly positive Δvalue estimates, promoting additional choice repetition biases on top of those driven by perception-choice learning.

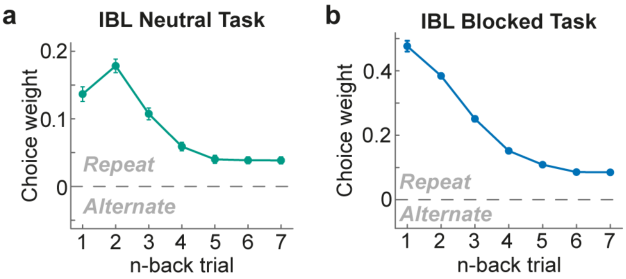

Supplementary Fig. 9 | Successful choice history kernels in the neutral and blocked task sessions of the International Brain Laboratory dataset.

a, History kernel comprising the past seven successful choice weights of the probabilistic choice model fit to the neutral sessions of the IBL dataset (n = 99 mice), before mice had experienced biased stimulus statistics (Methods, same as **Fig. 4c**). Mice exhibit a reduced 1- relative to 2-back choice weight. **b,** Same as in panel a, but for blocked task sessions of the same mice, in which stimuli were either more frequently presented on the left or the right side, in blocks of 20 to 100 trials. As expected from the high stimulus repetition probability within each block, mice exhibit overall stronger tendencies to repeat past choices and show a higher 1- compared to 2-back choice weight.

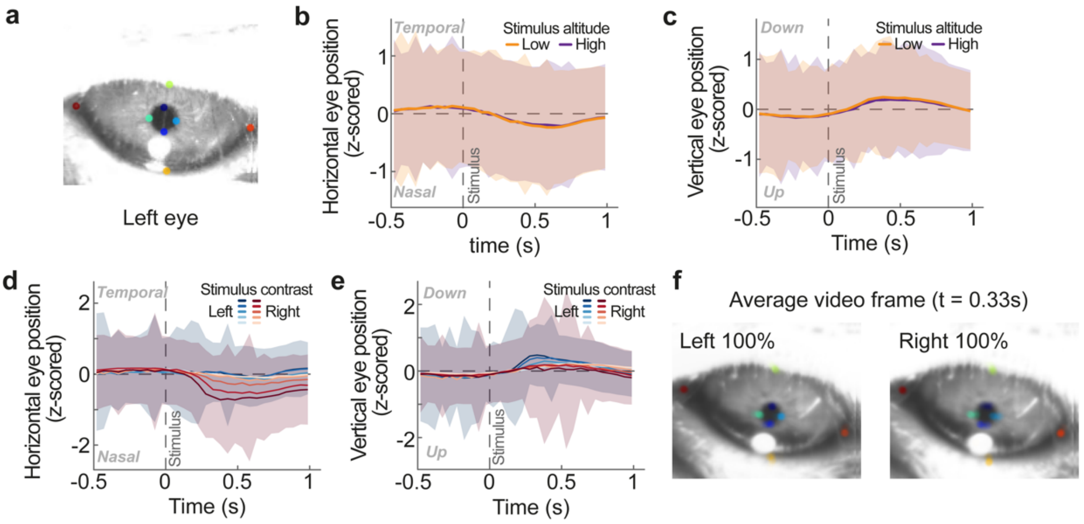

Supplementary Fig. 10 | Analysis of eye movements in experiment investigating spatially-specific sensory adaptation.
A, We used DeepLabCut to track the position of the left pupil of mice (*n* = 2) performing the visual decision-making task, investigating spatially-specific adaptation biases. **B, c,** Mice did not make differential eye movements in either horizontal (panel b) or vertical directions (panel c), when stimuli were presented in the lower (orange) or upper (purple) part of the visual field. Error regions in this and following panels depict standard deviation. **D,** Mice made very subtle but systematic horizontal eye movements in response to contralateral (right) stimuli, scaled by stimulus contrast (red lines). These eye movements were small in relation to the overall variability in eye position (shaded error bars). **E,** Mice made very subtle but systematic vertical eye movements in response to ipsilateral (left) stimuli (blue lines), scaled by stimulus contrast. **F,** Average video frame at 0.33 seconds after the onset of a left or right 100% contrast stimulus, for which differences in horizontal and vertical eye position were largest (see panels d and e). The difference in eye position is hardly detectable by eye, illustrating that differences in stimulus-related eye movements are miniscule.

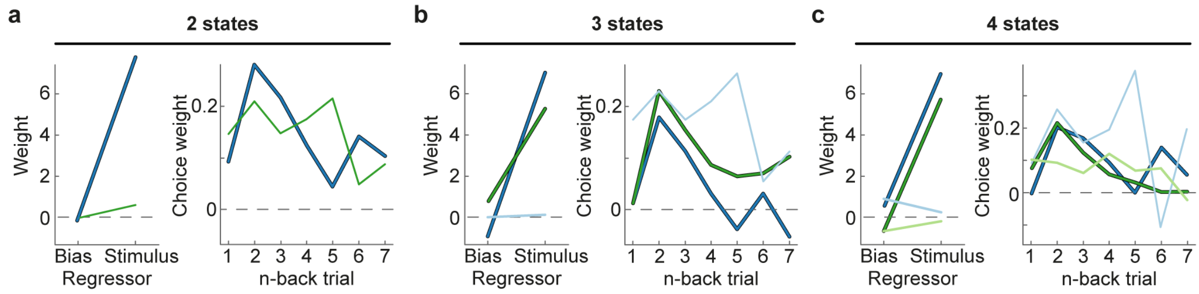

Supplementary Fig. 11 | Hidden Markov Models with choice history kernels show consistent history biases in task-engaged states.
The decreased tendency to repeat the 1- relative to 2-back choice in the neutral environment could be explained by mice pursuing two distinct decision-making strategies on distinct sets of trials, either alternating the previous choice while acting largely independent of the more long-term history, or repeating past choices monotonically weighted by their n-back position. To investigate this possibility, we conducted an analysis based on Hidden Markov Models[31] (HMMs). In particular, we fit HMMs with multiple states, and their state-specific Bernoulli Generalized Linear Models (GLMs) to our neutral environment task data. The GLMs consisted of a bias term, a stimulus regressor, and regressors for the past seven successful choices (choice history kernel). we fit separate HMMs with **(a)** 2, **(b)** 3, and **(c)** 4 states to the data. A model with 5 states returned a state with a large negative current stimulus weight, which is highly implausible and suggests overfitting. Importantly, if mice were to use a mixed strategy, we would expect to recover two clearly distinct choice history kernels – one kernel with monotonically decaying choice weights, and one kernel with a negative 1-back choice weight, and small 2- to 7-back choice weights. Instead, we found that all states with high current stimulus weights (bold

lines), indicating engagement with the task, were accompanied by the familiar choice history kernel with small positive 1-back weight, large positive 2-back weight, and a subsequent decay over 3- to 7-back trials. Only states with low stimulus weights (thin lines), indicating disengagement from the task, were accompanied by more flat choice repetition biases, consistent with the hypothesis that when mice disengage from the task, they default to repeating choices of the recent past (see **Supplementary Fig. 3**). Overall, the results of this analysis do not support the idea that the observed choice history kernel in the neutral environment is formed by a mixture of strategies on distinct sets of trials. Instead, it reinforces the conclusion that when mice are engaged with the neutral decision-making task, they tend to more strongly repeat the 2- rather than 1-back choice.

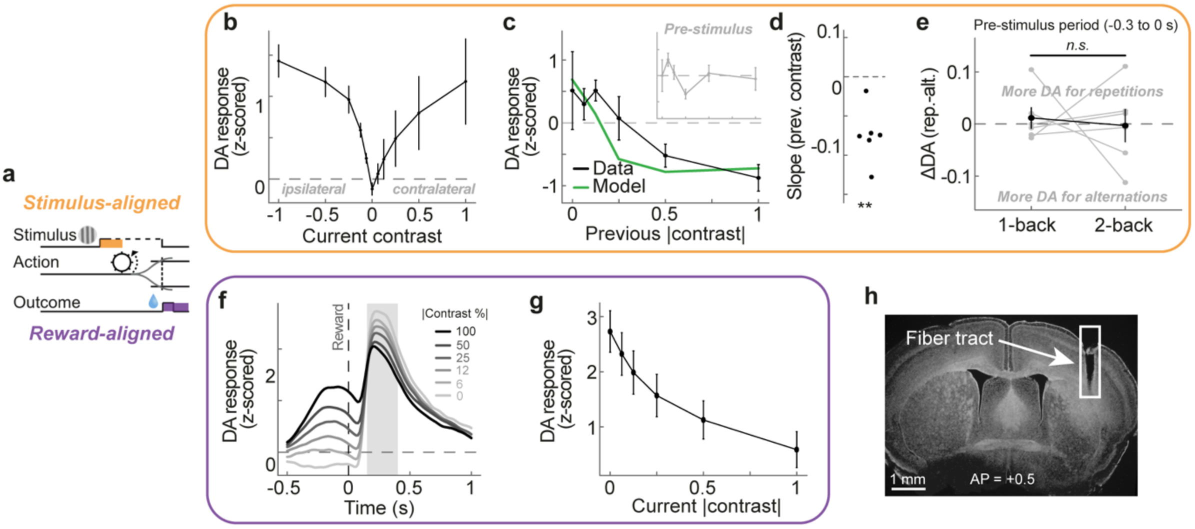

Supplementary Fig. 12 | Behavior and dopamine data of mice performing the visual decision-making task.
a, We time-locked dopamine responses to either the stimulus onset (orange) or reward onset (purple) of each successful trial. **b,** Group-average (*n* = 6 mice) stimulus-locked dopamine response (0.2 to 0.5s relative to stimulus onset) as a function of current stimulus contrast relative to the recorded hemisphere (x axis). Positive values indicate stimuli presented contralateral to the dopamine recording. The dopamine response positively scales with contrast, largely independent of stimulus side. **c,** Group-average stimulus-locked dopamine response (0.2 to 0.5s relative to stimulus onset; black line) as a function of previous absolute stimulus contrast (*x* axis, previous rewarded trials only). In the multi-trial reinforcement learning model, the expected reward value before outcome (*Q*, green line) is higher after previous successful choices based on low- rather than high-contrast stimuli, matching the empirical dopamine data. The relationship between previous contrast and dopamine is not evident in the pre-stimulus period (inset; -0.3 to 0s relative to stimulus onset; t(5) = -1.15, *p* = 0.30, two-sided t-test against zero of slope of linear model predicting dopamine by previous absolute contrast), suggesting that the effect is not due to a carryover of slow dopamine signals from the previous trial. **d,** The slope of a linear model predicting dopamine by previous absolute contrast shows a consistently negative relationship across mice (t(5) = -5.30, *p* = 0.003, two-sided t-test of slope against zero). **e,** Difference in pre-stimulus dopamine responses between repetitions and alternations of stimulus side (ΔDA) as a function of n-back trial (current and previous rewarded trials only). In contrast to the dopamine response after stimulus presentation (see **Fig. 6i**), there is no statistically significant difference in ΔDA between 1- and 2-back conditioned responses (t(5) = -0.30, *p* = 0.78, two-sided paired t-test). **f,** Group-average dopamine response, aligned to reward onset (gray dashed line), split by stimulus contrast (gray to black; correct trials only). Gray shaded area indicates the time period over which we averaged reward responses (0.15 to 0.4s after reward onset). The separation of dopamine responses before reward onset reflects stimulus- and choice-evoked dopamine release. **g,** Group-average dopamine following reward as a function of current absolute stimulus contrast. Responses are averaged over gray-shaded area in panel f, and baselined relative to the period immediately preceding the reward (-0.1 to 0s relative to reward onset). Dopamine scales negatively with current absolute stimulus contrast, opposite to pre-outcome responses (see **Fig. 6e**), and in line with a confidence-weighted dopaminergic prediction error. **h,** Example of post-mortem verification of fiber placement.

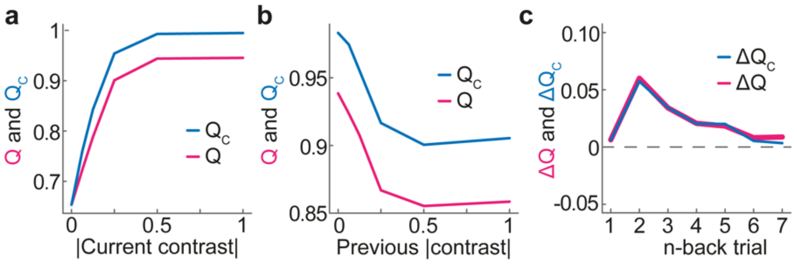

Supplementary Fig. 13 | The multi-trial model's expected reward value before and after committing a choice (*Q* and Q_C).
a, *Q* and Q_C similarly scale with the current absolute contrast. The expected reward value before the choice, *Q*, is computed by summing Q_L and Q_R weighted by the probability of making a left and right choice. The expected reward value after committing a choice is taken as either Q_L and Q_R for left and right choices, respectively. **b,** *Q* and Q_C similarly decrease with increasing absolute contrast of the previous trial (previous and current trials rewarded). **c,** Difference in *Q* (Q_C) between repetitions and alternations of stimulus side as a function of n-back trial (current and previous rewarded trials only).

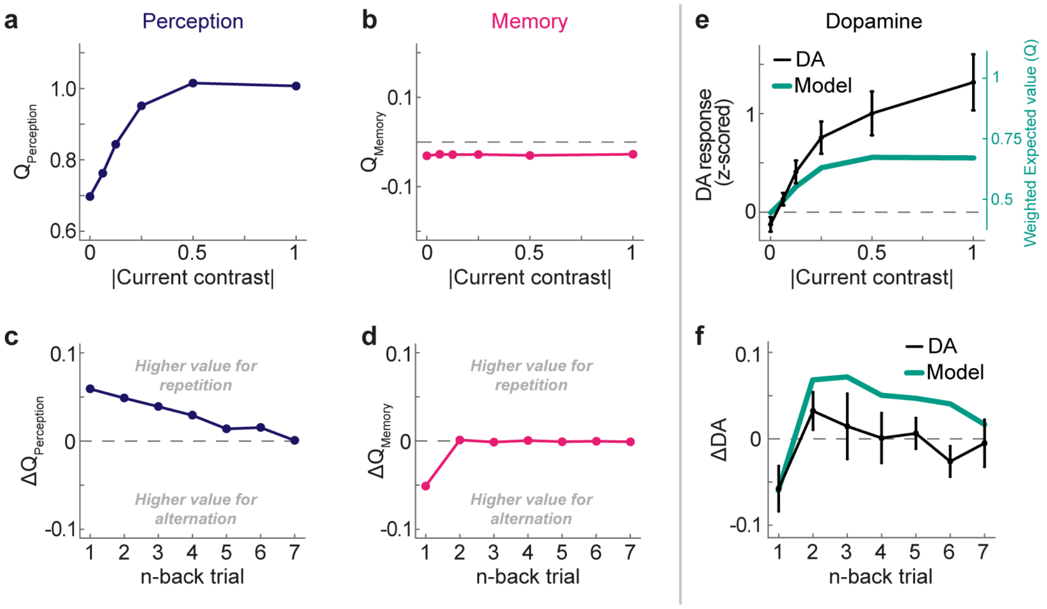

Supplementary Fig. 14 | Dopamine in the dorsolateral striatum may over-represent expectations calculated based on memory compared to perception.
a, The expected value based on the current visual stimulus increases with the absolute contrast of the current stimulus. **b,** The expected value based on the memory of the previous trial's choice does not depend on the current stimulus contrast. **c,** Difference in the perceptual component of expected value Q between repetitions and alternations of stimulus side ($\Delta Q_{Perception}$) as a function of n-back trial (current and previous rewarded trials only). Reward expectations based on the immediate sensory input initially favor stimulus repetitions and gradually decline towards zero. **d,** Same as in panel c, but for the memory component of the expected value. Reward expectations based on memory are higher for alternations compared to repetitions of the 1-back stimulus, and are zero otherwise, due to the limited memory of the multi-trial model. **e,** Average stimulus-evoked dopamine responses as a function of current absolute contrast (black; rewarded trials only), and **f,** difference in stimulus-evoked dopamine responses between repetitions and alternations of stimulus side (ΔDA) as a function of n-back trial (black; current and previous rewarded trials only). The trends in dopamine release are approximated by a weighted linear combination of perception-based (30%) and memory-based Q-values (70%; green lines in panels

e and f). The model's expected value was rescaled by a common scaling factor to plot the predictions for the dopamine data in both panels.

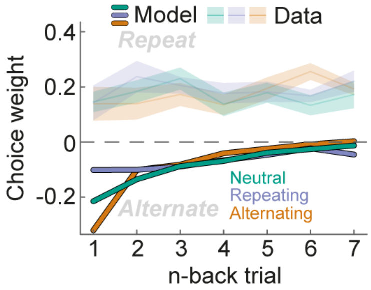

Supplementary Fig. 15 | Post-incorrect choice weights of mice (*n* = 10) and multi-trial belief state model
