# Supplementary material for: Temporal regularities shape perceptual decisions and striatal dopamine signals
Source: Nat Commun. 2024 Aug 17;15:7093. doi: 10.1038/s41467-024-51393-8 (PMC11330509; doi:10.1038/s41467-024-51393-8)
Supplement: Supplementary file 3 — Reporting Summary [file 41467_2024_51393_MOESM3_ESM.pdf]

nature portfolio

Corresponding author(s): Matthias Fritsche, Armin Lak

Last updated by author(s): Jul 3, 2024

Reporting Summary

Nature Portfolio wishes to improve the reproducibility of the work that we publish. This form provides structure for consistency and transparency in reporting. For further information on Nature Portfolio policies, see our Editorial Policies and the Editorial Policy Checklist.

Statistics

For all statistical analyses, confirm that the following items are present in the figure legend, table legend, main text, or Methods section.

n/a	Confirmed	
☐	☒	The exact sample size (n) for each experimental group/condition, given as a discrete number and unit of measurement
☐	☒	A statement on whether measurements were taken from distinct samples or whether the same sample was measured repeatedly
☐	☒	The statistical test(s) used AND whether they are one- or two-sided<br>*Only common tests should be described solely by name; describe more complex techniques in the Methods section.*
☐	☒	A description of all covariates tested
☐	☒	A description of any assumptions or corrections, such as tests of normality and adjustment for multiple comparisons
☐	☒	A full description of the statistical parameters including central tendency (e.g. means) or other basic estimates (e.g. regression coefficient) AND variation (e.g. standard deviation) or associated estimates of uncertainty (e.g. confidence intervals)
☐	☒	For null hypothesis testing, the test statistic (e.g. F, t, r) with confidence intervals, effect sizes, degrees of freedom and P value noted<br>*Give P values as exact values whenever suitable.*
☐	☒	For Bayesian analysis, information on the choice of priors and Markov chain Monte Carlo settings
☒	☐	For hierarchical and complex designs, identification of the appropriate level for tests and full reporting of outcomes
☒	☐	Estimates of effect sizes (e.g. Cohen's d, Pearson's r), indicating how they were calculated

Our web collection on statistics for biologists contains articles on many of the points above.

Software and code

Policy information about availability of computer code

Data collection	The experiments were controlled by freely available custom-made software, written in MATLAB (Mathworks): Bhagat, J., Wells, M. J., Harris, K. D., Carandini, M. & Burgess, C. P. Rigbox: An Open-Source Toolbox for Probing Neurons and Behavior. eNeuro 7, ENEURO.0406-19.2020 (2020)
Data analysis	Data analyses were performed with custom-made software written in Matlab 2020b, R (version 3.6.3), and Python 3.7. The GLM-HMM analysis was performed with the openly available glmhmm package (https://github.com/irisstone/glmhmm). The custom code developed in the current study is available in the Figshare database under accession code https://doi.org/10.6084/m9.figshare.24179829.

For manuscripts utilizing custom algorithms or software that are central to the research but not yet described in published literature, software must be made available to editors and reviewers. We strongly encourage code deposition in a community repository (e.g. GitHub). See the Nature Portfolio guidelines for submitting code & software for further information.

Data

Policy information about availability of data

All manuscripts must include a data availability statement. This statement should provide the following information, where applicable:

- Accession codes, unique identifiers, or web links for publicly available datasets
- A description of any restrictions on data availability
- For clinical datasets or third party data, please ensure that the statement adheres to our policy

The behavioral and photometry data generated in this study have been deposited in the Figshare database under accession code https://doi.org/10.6084/m9.figshare.24179829. The behavioral data of the International Brain Laboratory used in this study are available in the Figshare database under accession code https://doi.org/10.6084/m9.figshare.11636748.v7. Source data are provided with this paper.

Research involving human participants, their data, or biological material

Policy information about studies with human participants or human data. See also policy information about sex, gender (identity/presentation), and sexual orientation and race, ethnicity and racism.

Reporting on sex and gender	N/A
Reporting on race, ethnicity, or other socially relevant groupings	N/A
Population characteristics	N/A
Recruitment	N/A
Ethics oversight	N/A

Note that full information on the approval of the study protocol must also be provided in the manuscript.

Field-specific reporting

Please select the one below that is the best fit for your research. If you are not sure, read the appropriate sections before making your selection.

☒ Life sciences ☐ Behavioural & social sciences ☐ Ecological, evolutionary & environmental sciences

For a reference copy of the document with all sections, see nature.com/documents/nr-reporting-summary-flat.pdf

Life sciences study design

All studies must disclose on these points even when the disclosure is negative.

Sample size	The data of the behavioral experiment manipulating temporal regularities were collected from 10 mice. Of these mice, 3 animals also completed the experiment investigating sensory adaptation. Furthermore, we conducted dopamine recordings during perceptual decision-making in 6 mice. One of these mice also completed the sensory adaptation experiment. One mouse participated only in the sensory adaptation experiment. The sample size was determined based on previous studies investigating perceptual choice history biases in mice (Lak et al., 2020, Neuron).
Data exclusions	We excluded sessions exhibiting poor behavioral performance. In with the protocol of the International Brain Laboratory, we fit psychometric curves to the choice data of each session and excluded sessions when the absolute bias larger than 0.16, or either left or right lapse rates exceeded 0.2. We further excluded sessions in which the choice accuracy on easy 100% contrast trials was lower than 80%. This led to the exclusion of 56 out of 345 sessions (16%). Finally, we excluded trials in which the response time was longer than 12 seconds, thereby excluding 1,507 out of 128,490 trials (1.2%).
Replication	The increase in choice history weight from 1- to 2-back, a key signature of multi-trial learning, was replicated across three independent datasets, including openly available data collected across different laboratories (International Brain Lab). The increase in choice repetition after low- versus high-contrast rewarded trials was replicated across three independent datasets, including openly available data collected across different laboratories (International Brain Lab). All replication attempts were successful.
Randomization	For 8 out of 10 mice the order of environments with different temporal regularities was pseudo-randomized such that three consecutive Repeating or Alternating sessions were interleaved with two consecutive Neutral sessions. For the remaining two mice, the environments were presented in random order.
Blinding	The experimenters were not blind to the order of temporal regularities, as they had to specify the experimental parameters at the beginning of each session.

Reporting for specific materials, systems and methods

We require information from authors about some types of materials, experimental systems and methods used in many studies. Here, indicate whether each material, system or method listed is relevant to your study. If you are not sure if a list item applies to your research, read the appropriate section before selecting a response.

Materials & experimental systems

n/a	Involved in the study
☒	☐ Antibodies
☒	☐ Eukaryotic cell lines
☒	☐ Palaeontology and archaeology
☐	☒ Animals and other organisms
☒	☐ Clinical data
☒	☐ Dual use research of concern
☒	☐ Plants

Methods

n/a	Involved in the study
☒	☐ ChIP-seq
☒	☐ Flow cytometry
☒	☐ MRI-based neuroimaging

Animals and other research organisms

Policy information about studies involving animals; ARRIVE guidelines recommended for reporting animal research, and Sex and Gender in Research

Laboratory animals	C57BL/6J mouse (aged 10-30 weeks) from Charles River UK.
Wild animals	The study did not involve wild animals
Reporting on sex	male
Field-collected samples	The study did not involve samples collected from the field.
Ethics oversight	All experiments were conducted according to the UK Animals Scientific Procedures Act (1986) under appropriate project and personal licenses by the UK Home Office.

Note that full information on the approval of the study protocol must also be provided in the manuscript.
